# Posttraumatic Haematuria with Pseudorenal Failure: A Diagnostic Lead for Intraperitoneal Bladder Rupture

**DOI:** 10.1155/2016/4521827

**Published:** 2016-07-28

**Authors:** Ketan Vagholkar, Suvarna Vagholkar

**Affiliations:** Department of Surgery, D.Y. Patil University, School of Medicine, Navi Mumbai 400706, India

## Abstract

Bladder rupture is a very morbid injury following blunt or penetrating lower abdominal trauma. Prompt diagnosis is crucial to initiate optimal treatment. Intraperitoneal bladder rupture is associated with haematuria and biochemical features of renal failure. Cystogram is diagnostic. Immediate open surgical repair is the main stay of treatment. A case of intraperitoneal rupture diagnosed preoperatively by the presence of haematuria and pseudorenal failure is presented to highlight the association of posttraumatic haematuria and pseudorenal failure in such injuries.

## 1. Introduction

Intraperitoneal bladder rupture is a morbid injury associated with both blunt and penetrating trauma. Penetrating injury makes the diagnosis of bladder rupture much easier. However blunt injury with no associated pelvic fracture makes diagnosis of intraperitoneal rupture very difficult. The haematological changes of acute renal failure in such patients usually mislead the attending surgeon in arriving at diagnosis [1].

A case of intraperitoneal bladder rupture following blunt abdominal injury accompanied with features of acute renal failure best described as acute pseudorenal failure and haematuria is presented with a view to highlight this association.

## 2. Case Report

A 27-year-old male patient was admitted to our surgical facility with history of a fall from a bus while being under influence of alcohol.

The patient was taken to nearest hospital from where he was referred to our surgical unit two days after the injury.

On admission patient was fully conscious and gave history of an alcohol binge following which he had a fall from a moving bus. He had severe haematuria on admission. There were no external marks of any other injury. Patient had no other comorbid medical conditions.

On examination patient had a pulse of 96 beats/min, blood pressure was 120/70 mm of Hg, and he had pallor.

Perabdominal examination did not reveal any distension, tenderness, rebound tenderness, guarding, or rigidity. There was no external evidence of any injury in the thoracoabdominal region. The genitalia were normal.

Patient passed urine which showed gross haematuria. As patient had passed urine by himself with no surrounding soft tissue swelling a trial of catheterisation was given. The catheter could be passed in smoothly without any resistance. Approximately 500 cc of frank haematuric urine was drained.

**Figure 1:**
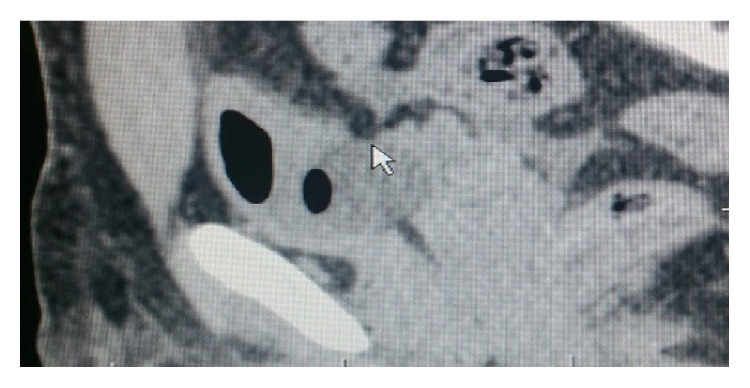
Plain CT scan showing the rent in the bladder marked by the arrow.

**Figure 2:**
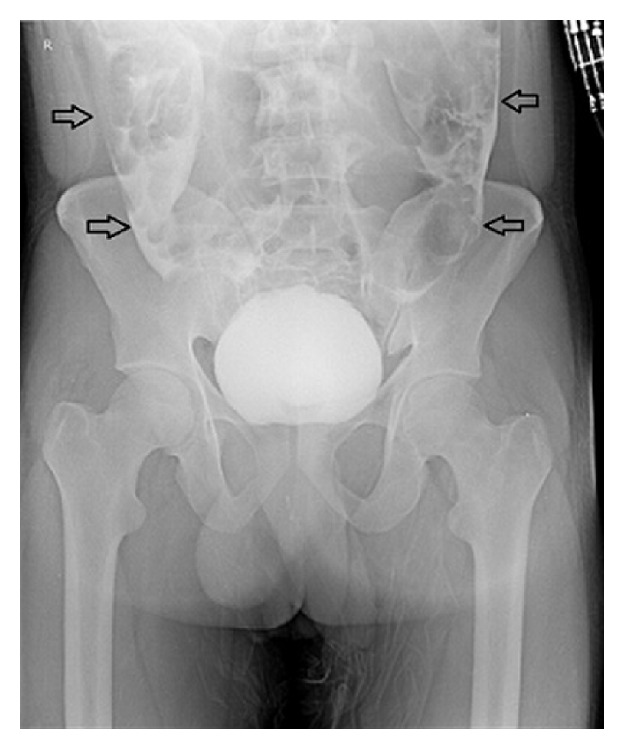
Cystogram showing extravasation of the contrast into the peritoneal cavity marked by arrows.

**Figure 3:**
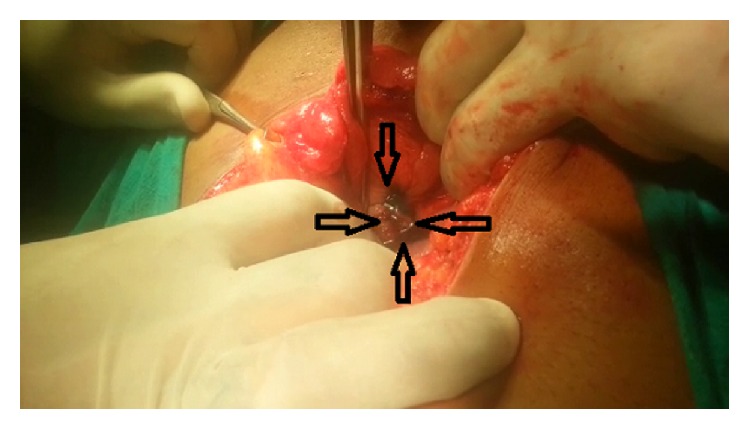
Intraoperative confirmation of the rent in the posterior wall marked by black arrows after instilling methylene blue per urethral.

Blood investigations revealed a haemoglobin of 16.5 gm with haematocrit of 40. Total count was 6500, blood urea nitrogen was 45 mgm%, serum creatinine was 7.4 mg%, and serum electrolytes were within normal range. Intravenous resuscitation was given with normal saline causing clearing of haematuria with decrease in the tachycardia. Patient underwent plain CT scan of abdomen which revealed normal upper abdominal viscera. However the pelvis revealed a suspicious breach in the posterior wall of the urinary bladder (Figure 1). Plain cystogram obtained by instilling 300 cc of diluted contrast revealed gross leaking of contrast into the general peritoneal cavity (Figure 2). A repeat serum creatinine was done at this stage and showed significant fall and was reduced to 2.5 mg%. Patient underwent exploratory laparotomy. At laparotomy methylene blue was instilled into bladder through the per urethral catheter. A large rent in the posterior wall of bladder measuring 3 cm horizontally and 1 cm vertically was identified (Figure 3). The rent was sutured in two layers by absorbable suture material with adequate drainage of bladder by both suprapubic and per urethral catheter as well. Postoperative recovery was uneventful. Postoperative serum creatinine was 0.8 mg%.

## 3. Discussion

Diagnosis of rupture of the urinary bladder is a challenging issue. The etiology may vary from trauma either blunt or penetrating to spontaneous rupture. Depending upon the state of the bladder it may rupture intraperitoneally or extraperitoneally. The urinary bladder assumes a variable position in the abdominal cavity depending upon volume of its content. When it is empty it lies deeply in pelvis, when full becomes intraperitoneal organ.

Trauma to the lower abdominal wall while the bladder is fully distended can cause damage ranging from a contusion to frank rupture. If there is no associated injury such as pelvic fracture the abdominal signs may be subtle. This can be misleading resulting in failure to clinically diagnose a serious bladder injury. However severe haematuria should raise a strong suspicion of a bladder rupture.

Urinary extravasation into the free intraperitoneal cavity in large volume can lead to diffusion of solutes and toxins excreted in urine along the concentration gradient, a phenomenon described as reverse autodialysis [1, 2]. The more the delay in presentation and diagnosis, the more severe the biochemical abnormalities [3].

This was typically seen in the case presented where serum creatinine on admission was high and showed a steady decline to normalcy after drainage and repair. Therefore in a case of posttraumatic haematuria one needs to be aware of the fact that features of acute renal failure are seen. The serum creatinine levels are usually very high. However the other renal parameters are surprisingly normal despite a very high creatinine. This phenomenon is best described as acute pseudorenal failure [4]. A combination of haematuria with features of acute pseudorenal failure should therefore raise a strong suspicion of intraperitoneal bladder rupture.

The diagnostic imaging modality is plain cystogram, which typically reveals the extravasation of the contrast into the general peritoneal cavity as was seen in the case presented (Figure 2). CECT can also be done. It will not only reveal the bladder rupture but also reveal the status of the bony pelvis and other abdominal viscera including vascular injuries. Ultrasound evaluation will reveal free fluid in the peritoneal cavity. An ultrasound guided aspiration of fluid will help in confirming the diagnosis as well as determining the choice of antibiotics based on culture studies of the urine aspirate.

However a plain cystogram is superior to a CT cystogram or an ultrasound [5]. This has been revealed in various studies. However CECT is indicated in most cases of both blunt and penetrating abdominal trauma to rule out concomitant visceral injury which could otherwise be missed [5–7].

Surgery is the main stay of treatment in intraperitoneal bladder rupture [8, 9]. Identification of the rent with a two-layered closure by absorbable suture material followed by adequate drainage of bladder is best standard of care. In rare cases of smaller leaks seen in patients with severe comorbidities such as diabetes or COPD a trial of conservative treatment can be contemplated.

## 4. Conclusion

Frank haematuria following blunt abdominal injury associated with biochemical features of renal failure should strongly raise the suspicion of intraperitoneal bladder rupture.

A plain cystogram plate is both diagnostic and confirmatory.

Prompt surgical exploration with repair and bladder drainage is the mainstay of treatment.
